# Involvement of flocculin in negative potential-applied ITO electrode adhesion of yeast cells

**DOI:** 10.1093/femsyr/fov064

**Published:** 2015-07-17

**Authors:** Sumihiro Koyama, Taishi Tsubouchi, Keiko Usui, Katsuyuki Uematsu, Akihiro Tame, Yuichi Nogi, Yukari Ohta, Yuji Hatada, Chiaki Kato, Tetsuya Miwa, Takashi Toyofuku, Takehiko Nagahama, Masaaki Konishi, Yuriko Nagano, Fumiyoshi Abe

**Affiliations:** 1 Department of Marine Biodiversity Research, Japan Agency for Marine-Earth Science and Technology, 2-15 Natsushima-cho, Yokosuka, Kanagawa 237-0061, Japan; 2 Research and Development Center for Marine Biosciences, Japan Agency for Marine-Earth Science and Technology, 2-15 Natsushima-cho, Yokosuka, Kanagawa 237-0061, Japan; 3 Department of Marine Science, Marine Works Japan Ltd., 3-54-1 Oppamahigashi, Yokosuka 237-0063, Japan; 4 Marine Technology and Engineering Center, Japan Agency for Marine-Earth Science and Technology, 2-15 Natsushima-cho, Yokosuka, Kanagawa 237-0061, Japan; 5 Department of Foods and Human Nutrition, Notre Dame Seishin University, 2-16-9 Ifuku-cho, Kita-ku, Okayama 700-8516, Japan; 6 Department of Biotechnology and Environmental Chemistry, Kitami Institute of Technology, 165 Koen-cho, Kitami, Hokkaido 090-8507, Japan; 7 Department of Chemistry and Biological Science, College of Science and Engineering, Aoyama Gakuin University, 5-10-1 Fuchinobe, Chuo-ku, Sagamihara 252-5258, Japan

**Keywords:** *Saccharomyces cerevisiae*, Flo10, electrical attachment, potential-controlled electrode, indium tin oxide, single-cell cultivation

## Abstract

The purpose of this study was to develop novel methods for attachment and cultivation of specifically positioned single yeast cells on a microelectrode surface with the application of a weak electrical potential. *Saccharomyces cerevisiae* diploid strains attached to an indium tin oxide/glass (ITO) electrode to which a negative potential between −0.2 and −0.4 V vs. Ag/AgCl was applied, while they did not adhere to a gallium-doped zinc oxide/glass electrode surface. The yeast cells attached to the negative potential-applied ITO electrodes showed normal cell proliferation. We found that the flocculin *FLO10* gene-disrupted diploid BY4743 mutant strain (*flo10*Δ /*flo10*Δ) almost completely lost the ability to adhere to the negative potential-applied ITO electrode. Our results indicate that the mechanisms of diploid BY4743 *S. cerevisiae* adhesion involve interaction between the negative potential-applied ITO electrode and the Flo10 protein on the cell wall surface. A combination of micropatterning techniques of living single yeast cell on the ITO electrode and omics technologies holds potential of novel, highly parallelized, microchip-based single-cell analysis that will contribute to new screening concepts and applications.

## Abstract (precis)

Yeast *Saccharomyces cerevisiae* were selectively attached on the negative potential-applied indium tin oxide/glass electrode. Mechanisms of the yeast cell attachment involve Flocculin Flo10 proteins.

## Graphical abstract

**Graphical Abstract Figure.**
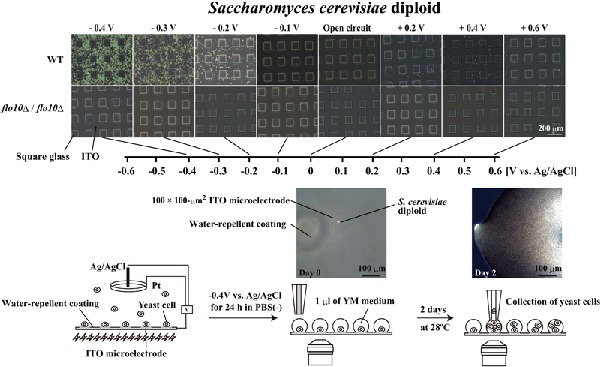
Yeast *Saccharomyces cerevisiae* were selectively attached on the negative potential-applied indium tin oxide/glass electrode. Mechanisms of the yeast cell attachment involve Flocculin Flo10 proteins.

## INTRODUCTION

The first complete genome sequence of the yeast *Saccharomyces cerevisiae* was published (Goffeau *et al.* 1996), enabling a wide variety of genetic engineering techniques and the strict functional analyses of proteins (Giaever *et al.* 2002; Shibasaki, Maeda and Ueda 2009; Breker, Gymrek and Schuldiner 2013). A heterozygous diploid mutant collection of approximately 6000 strains of *S. cerevisiae*, in each of which one copy of a single gene is deleted, is commercially available. With this collection, it is possible to evaluate the role of each gene product in the response of cells to a drug (Lum *et al.* 2004; Parsons *et al.* 2004, 2006; Roberge 2008). Drug-induced haploinsufficiency refers to the situation where a heterozygous diploid mutant is more sensitive to a drug than is the wild-type strain (Roberge 2008). Drug-induced haploinsufficiency profiling (HIP)/homozygous profiling (HOP) assay was one of the first assays to take advantage of parallelized growth strategy (Smith *et al.* 2010). The HIP/HOP assay has been applied to identify the protein targets in numerous drugs and successfully employed in industry (Smith *et al.* 2010; Giaever and Nislow 2014).

Micropatterning techniques of single animal cells have been developed during the past decade to assess and monitor cell physiology and intracellular protein functions, to analyze and evaluate chemical reactions within cells such as toxicity testing and the identification of drug targets, and to screen for novel biotechnological products such as genetically modified enzymes and biomolecules (Whitesides *et al.* 2001; Jiang *et al.* 2003; Gray *et al.* 2004; Albrecht *et al.* 2005; Xu *et al.* 2005; Veiseh and Zhang 2006; Rundqvist *et al.* 2007; Yap and Zhang 2007; Ino *et al.* 2009; Robertus, Browne and Feringa 2010; Koyama 2011). Micropatterning techniques based on animal cell–extracellular matrix interactions utilize the characteristics of cell adhesion activity to extracellular matrix proteins. Although some yeasts such as *S. cerevisiae*, *Candida albicans* and *C. glabrata* have the ability to adhere to plastic surfaces (Reynolds and Fink 2001; Verstrepen and Klis 2006; Mortensen *et al.* 2007; Van Mulders *et al.* 2009; Kucharíková *et al.* 2011), yeast cell adhesion to plastic substrates is believed to depend on hydrophobic interaction (Van Mulders *et al.* 2009; Alsteens *et al.* 2013). Therefore, the attachment of yeast cells to a transparent substrate surface in densely positioned single-cell array configurations such as animal cell microarray techniques has not been reported because of the difficulty in modifying single-cell yeast attachment. Previously, we reported that the hydrophilicity of an optically transparent electrode surface was modulated by weak potential applications (Koyama 2011). Both the constant and high-frequency wave potentials increased hydrophilicity on the electrode surface (Koyama 2011). The cultivation of heterozygous diploid mutant single yeast cells attached to a potential-controlled microelectrode array will allow iterative screening tests of numerous pharmaceutical compound candidates when the compound-containing media are replaced. Moreover, the yeast cell patterning techniques enable the performance of novel, highly parallelized, microchip-based single-cell analysis that will contribute to new screening concepts and applications when only small amounts of yeast are available.

In our previous study, living soil and sediment microorganisms suspended in nonnutritive media such as PBS(−) and artificial seawater were selectively attached to negative potential-applied indium tin oxide/glass (ITO) electrode regions against gravitational force (Koyama *et al.* 2013). Microorganisms suspended in Luria–Bertani medium and glucose solution were not attracted to the ITO electrode. Dead microorganisms were not attracted to the ITO electrode, either. Microorganisms such as *Escherichia coli* recognized small regions of the negative applied-potential microelectrode and selectively attached to the 5-μmϕ circular microelectrode array, however (Koyama *et al.* 2013). When we applied the electrical retrieval method to separate the microorganisms from sediment and soil particles, bacteria belonging to 19 phyla and 23 classes were collected without undesirable high-molecular-weight contaminants such as humic acids (Koyama *et al.* 2013). In the present study, we developed novel methods for the attachment and cultivation of specifically positioned single yeast cells on a microelectrode surface with the application of a weak negative electrical potential.

## MATERIALS AND METHODS

### Yeast strains and growth conditions

The yeasts used in this study are listed in Table 1. All were cultured with shaking (120–150 rpm) at 28ºC for 24 h in yeast mold (YM) medium (Difco YM Broth, Becton Dickinson and Company, Sparks, MD, USA).

**Table 1.** *Saccharomyces cerevisiae* strains used in this study.

Strains	Genotype/description	Source
BY4741 (wild type)	*MATa his3Δ1 leu2Δ0 met15Δ0 ura3Δ0*	Brachmann *et al.* (1998)
		Giaever *et al.* (2002)
BY4742 (wild type)	*MATα his3Δ1 leu2Δ0 lys2Δ0 ura3Δ0*	Brachmann *et al.* (1998)
		Giaever *et al.* (2002)
BY4743 (wild type)	*MATa/α his3Δ1/his3Δ1 leu2Δ0/leu2Δ0*	Brachmann *et al.* (1998)
	*met15Δ0/+ lys2Δ0/+ ura3Δ0/ura3Δ0*	Giaever *et al.* (2002)
BY4741 (*flo1Δ*)	*flo1Δ* mutant strain of BY4741	Giaever *et al.* (2002)
BY4742 (*flo1Δ*)	*flo1Δ* mutant strain of BY4742	Giaever *et al.* (2002)
BY4743 (*flo1Δ /flo1Δ*)	*flo1Δ /flo1Δ* mutant strain of BY4743	Giaever *et al.* (2002)
BY4741 (*flo10Δ*)	*flo10Δ* mutant strain of BY4741	Giaever *et al.* (2002)
BY4742 (*flo10Δ*)	*flo10Δ* mutant strain of BY4742	Giaever *et al.* (2002)
BY4743 (*flo10Δ /flo10Δ*)	*flo10Δ /flo10Δ* mutant strain of BY4743	Giaever *et al.* (2002)
YPH499 (wild type)	*MATa ura3–52 lys2–801 ade2–101*	Sikorski and Hieter (1989)
	*trp1-Δ63 his3-Δ200 leu2-Δ1*	
YPH500 (wild type)	*MATα ura3–52 lys2–801 ade2–101*	Sikorski and Hieter (1989)
	*trp1-Δ63 his3-Δ200 leu2-Δ1*	
YPH501 (wild type)	*MATa/α ura3–52/ura3–52 lys2–801/lys2–801*	Sikorski and Hieter (1989)
	*ade2–101/ade2–101 trp1-Δ63/trp1-Δ63*	
	*his3-Δ200/his3-Δ200 leu2-Δ1/leu2-Δ1*	

### Electrode preparation

**Figure 1.**
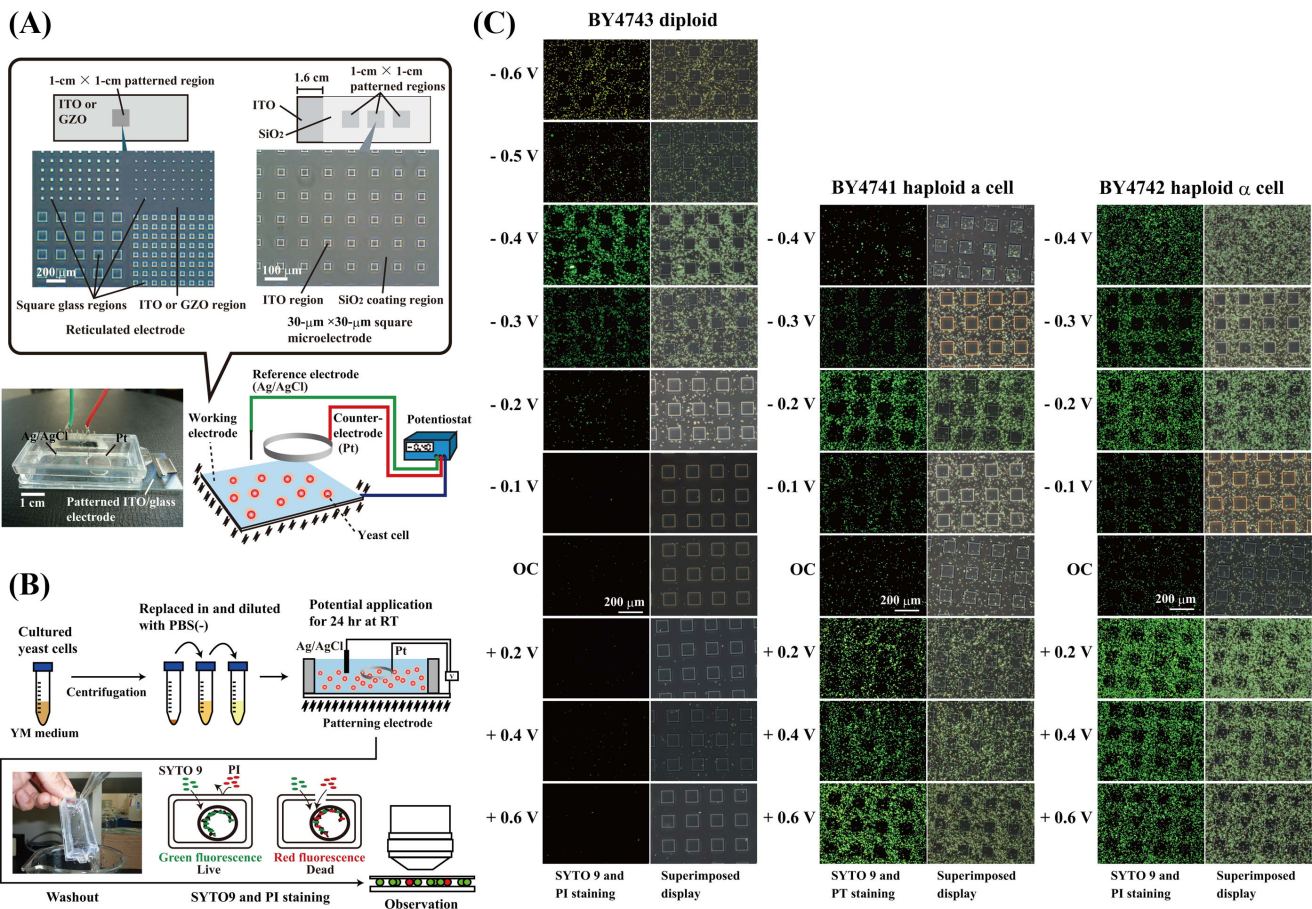
Attachment of *S. cerevisiae* yeast cells to the potential-applied ITO electrode. (**A**) An optically transparent working electrode was placed on bottom of a chamber device with a counter- (Pt) and a reference (Ag/AgCl) electrode. The electrode potential is controlled with an Ag/AgCl reference electrode through a potentiostat. (**B**) Schematic illustration of the electrical attachment method for yeasts is shown. 1 × 10^7^ cells/5 ml in PBS(−) of each yeast strain was seeded into the three-electrode chamber device. (**C**) The *S. cerevisiae* cells attached to the constant potential-applied patterned ITO electrodes in PBS(−) after 24 h at RT. PI, propidium iodide.

**Figure 2.**
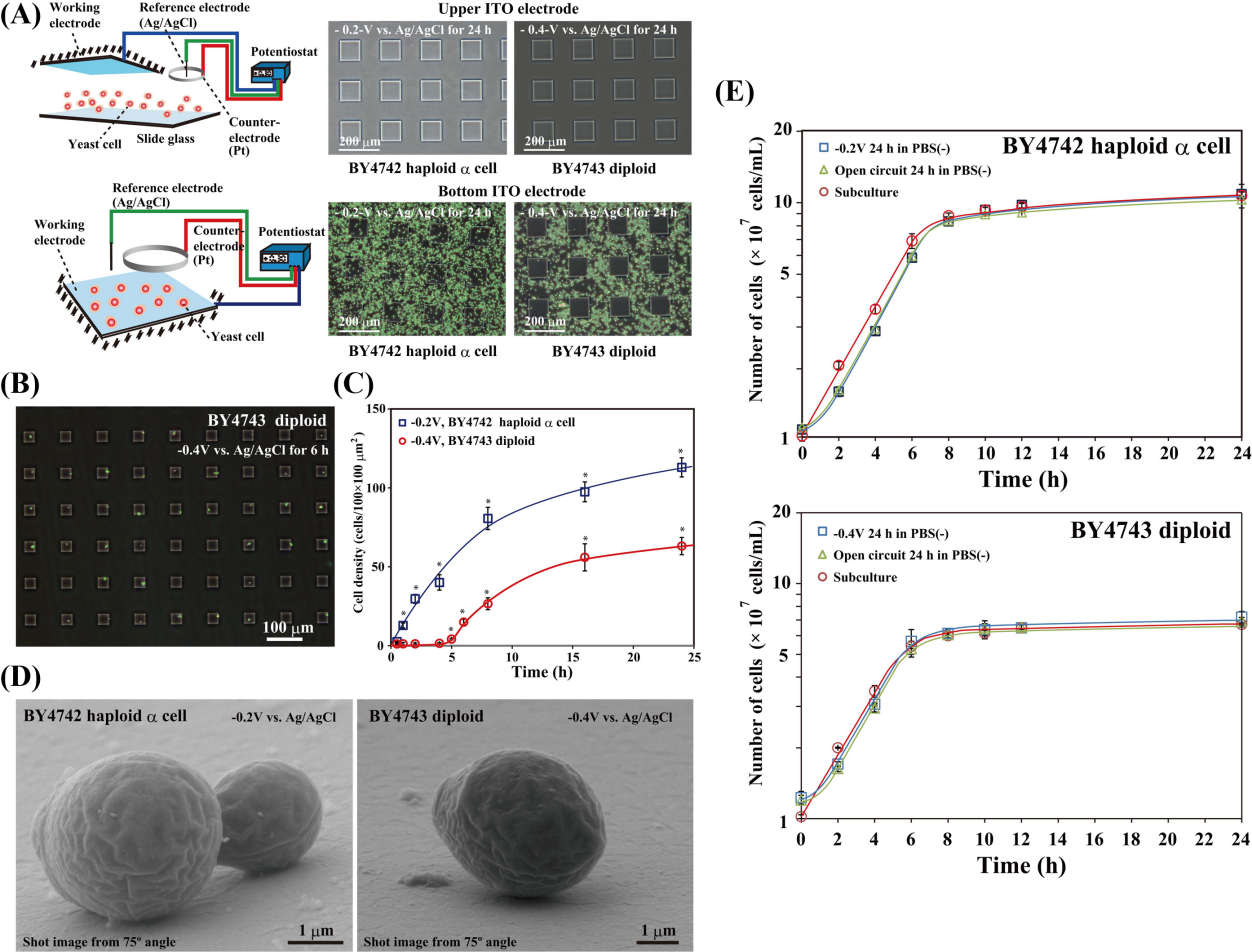
Attachment of *S. cerevisiae* strains to the potential-applied ITO electrode. (**A**) Strains BY4742 and BY4743 on either the top or bottom of the patterned ITO electrode surface in PBS(−) after 24 h at RT. (**B**) The diploid BY4743 *S. cerevisiae* strain attached to 30 × 30 μm^2^ microelectrodes to which a −0.4 V vs. Ag/AgCl potential was applied in PBS(−) at RT. (**C**) Time course of haploid and diploid *S. cerevisiae* strain attachment to the electrode with either a −0.2 V or a −0.4 V vs. Ag/AgCl applied potential in PBS(−) at RT. The values are mean ± SEM (*n* = 8). *P < 0.001 compared with 0.5 h. (**D**) SEM images of haploid and diploid *S. cerevisiae* strains attached to the reticulated ITO electrode to which either a −0.2 V or a −0.4 V vs. Ag/AgCl potential was applied in PBS(−) for 24 h at RT. The images were obtained from a 75º angle. (**E**) Cell growth comparison of *S. cerevisiae* cells from the ITO electrode. The haploid and diploid yeast cell growth was monitored after cell scraping or subculture. Either an OC, a −0.2 V vs. Ag/AgCl constant potential, or −0.4 V vs. Ag/AgCl constant potential was applied for 24 h in PBS(−) at RT. The values are mean ± SEM (*n* = 4).

Patterned working electrodes (Figs 1A and 2A and B) were constructed by vacuum evaporation of either ITO (In_2_O_3_; <10 Ω cm^−2^) or gallium-doped zinc oxide (GZO; <10 Ω cm^−2^) and an insulator of silicon dioxide (SiO_2_) onto 76 × 26 mm^2^ silica glass plates (1.1 mm thick) (Geomatec Co., Ltd, Yokohama, Japan). The reticulated ITO electrode with arrayed square glass regions (Figs 1A and 2A) was described elsewhere (Koyama *et al.* 1997, 2013; Koyama 2011). The 30 × 30 μm^2^ microelectrode was formed by the plane ITO electrode fabricated with a coating of SiO_2_ (Fig. 2B). The plastic chamber section of a Lab-tek II chamber slide system (Cat. 154453, NalgeNunc International, Naperville, IL, USA) was glued to the patterned working electrode with silicon bonding. A 12-mmϕ section of both the Pt ring counter electrode and Ag/AgCl reference electrode was placed on the plastic lid of the chamber slide system. The Pt counter electrode was positioned directly above the patterning region of the working electrode. For electrical yeast attachment experiments against gravitational force, we placed the patterned working electrode on top of the chamber device (Fig. 2A; Koyama *et al.* 2013). A large electrode chamber device (Koyama *et al.* 2013) was used for comparative analysis of *S. cerevisiae* cell growth. The microelectrode array (Fig. 4A) was formed by the plane ITO electrode fabricated with SiO_2_ and water-repellent coating (contact angle of water droplet is about 115º). A silicon rubber plate 90 × 90 mm^2^ and 5 mm thick with a hollow interior measuring 80 × 80 mm^2^ was glued to a 100 × 90 mm^2^ microelectrode array with silicon bonding. The microelectrode array was placed on the bottom of the large electrode chamber device and housed in a sterile square plastic dish. Sections of the Pt ring counter electrode (30 mmϕ) and Ag/AgCl reference electrode were placed on the plastic lid of the square plastic dish.

**Figure 3.**
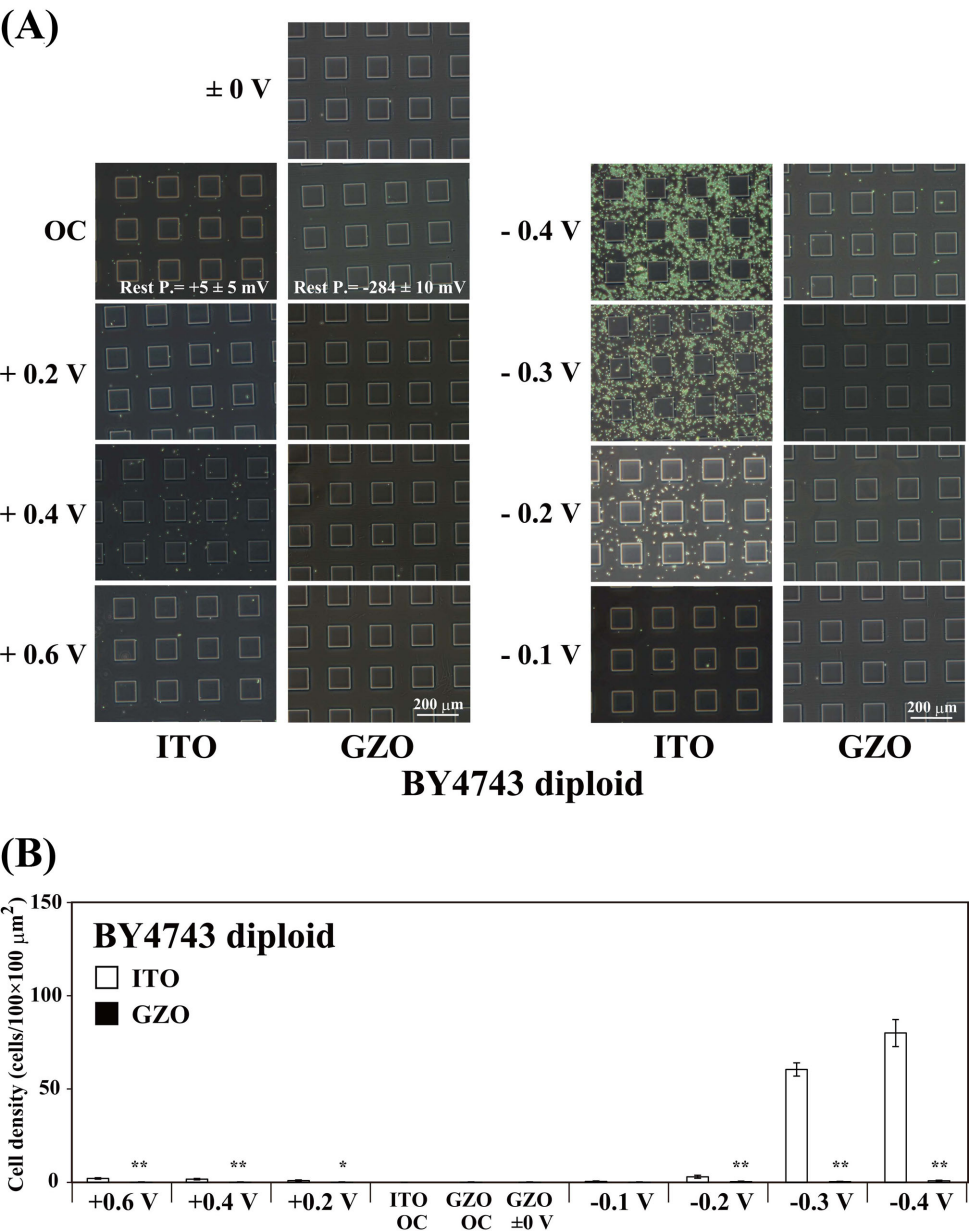
Electrode surface recognition properties of the diploid *S. cerevisiae* strain. (**A**) Distribution patterns of strain BY4743 on the potential-applied ITO and GZO electrodes in PBS(−) after 24 h at RT. (**B**) Cell densities of strain BY4743 on the potential-applied ITO and GZO electrodes in PBS(−) after 24 h at RT. A potential between −0.4 V and +0.6 V vs. Ag/AgCl was applied to either an ITO or a GZO electrode for 24 h in PBS(−) at RT. The values are mean ± SEM (*n* = 8). *P < 0.05, **P < 0.001 compared with the ITO electrode. The resting potentials of the GZO and ITO electrodes in PBS(−) at RT were −284 ± 10 mV vs. Ag/AgCl (mean ± SEM; *n* = 12) and +5 ± 5 mV vs. Ag/AgCl (mean ± SEM; *n* = 104), respectively.

**Figure 4.**
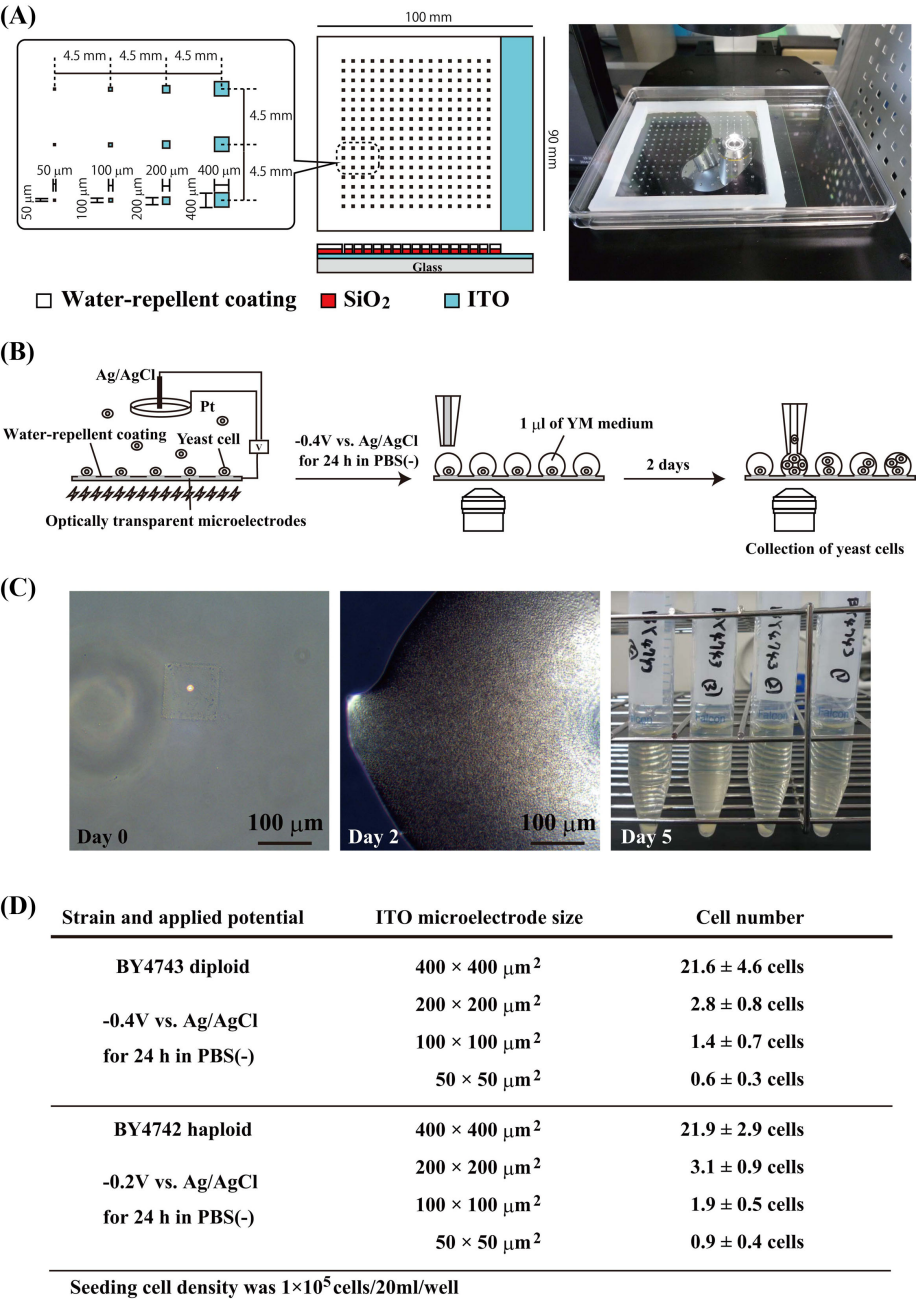
Single yeast cell cultivation on the ITO microelectrode array. (**A**) Schematic illustration and photograph of an ITO microelectrode array. (**B**) Schematic illustration of the electrical attachment and cultivation method for single yeast cells is shown. (**C**) Single-cell diploid BY4743 cultivation on a 100 × 100 μm^2^ microelectrode. The diploid BY4743 single cells attached to a small region of the ITO microelectrode to which a −0.4 V vs. Ag/AgCl potential was applied at RT in PBS(−). After the removal of PBS(−), 1 μL of YM medium was added to each ITO microelectrode region and then incubated for 2 days at 28ºC. The proliferating yeast cells on the microelectrode were transferred to 15-ml centrifuge tubes and cultured for a further 3 days. (**D**) Cell number of the electrically attached diploid and haploid yeast cells on a small square region of the ITO microelectrode. The values are mean ± SEM (*n* = 8).

The working electrodes were sonicated in ultrapure water for 5 min and immersed in 1 M NaOH for 5 min to remove any unwanted deposits and then washed with ultrapure water and dried. The three electrode chambers were subsequently irradiated with UV light (254 nm, 30W, GL30, Toshiba lighting and technology corporation, Kanagawa, Japan) in a biological safety cabinet for 5 min for sterilization.

### Potential application

The constant potential was modulated with a potentiostat and applied to the patterned working electrode using the Ag/AgCl reference electrode and the Pt counter electrode (Figs 1, 2 and 4; Koyama *et al.* 1997, 2013; Koyama 2011). For the attachment of the yeast cells to the patterned working electrode regions (Figs 1A, 2A and B, and 4A), a constant potential was applied to the electrode at room temperature (RT). The cultured yeast cells were collected by centrifugation for 2 min at 2150 × *g* and 4ºC and suspended in either Dulbecco's calcium magnesium-free phosphate-buffered saline [PBS(−); Wako, Osaka, Japan], 280 mM mannose, 280 mM glucose, 280 mM maltose, 280 mM sucrose or 280 mM galactose solution. In the electrical attachment experiments with dead yeast cells, the pellets were resuspended in 70% EtOH with vortexing and incubated for 1 h at 60ºC. After 70% EtOH fixation, the yeast cells were centrifuged for 2 min at 2150 × *g* and 4ºC and then replaced in PBS(−). The yeast cell suspensions were diluted to a concentration of 1 × 10^7^ cells/5 ml and poured into the three-electrode chamber system (Fig. 1B).

### Cell growth comparison analysis

The yeast cells were suspended in PBS(−) at RT, diluted to a concentration of 2 × 10^8^ cells/12.5 ml and poured into the large electrode chamber device. When using the haploid yeast strain BY4742, either a −0.2 V vs. Ag/AgCl constant potential or a resting potential (open circuit, OC) was applied to the large electrode for 24 h at RT in PBS(−). When using the diploid yeast strain BY4743, either a −0.4 V vs. Ag/AgCl constant potential or a resting potential (OC) was applied to the large electrode for 24 h at RT in PBS(−). After 24 h, the electrode was washed with PBS(−) at RT, and the yeast cells attached to the electrode were detached with a cell scraper. The detached yeast cells were collected with the cell scraper, resuspended in fresh YM medium and diluted to a concentration of 1 × 10^7^ cells ml^−1^. In the positive control experiments, the yeast cells were cultured with shaking (140 rpm) at 28ºC for 24 h and then diluted to a concentration of 1 × 10^7^ cells ml^−1^ in fresh YM medium. One milliliter of each yeast cell suspension was transferred to a 2-ml cryotube (Greiner Bio-One GmbH, Frickenhausen, Germany). The yeast cells in the cryotubes were shaken (140 rpm) and cultured at 28ºC for 2, 4, 6, 8, 10, 12 and 24 h, respectively. The cultured yeast cell density was measured with a hemacytometer. Measurement of the yeast cell density was repeated four times, and data were expressed as the mean of two independent experiments.

### Single yeast cell cultivation on a microelectrode array

The yeast cell suspensions were replaced in PBS(−) at RT, diluted to a concentration of 1 × 10^5^ cells/20 ml and poured into the microelectrode array chamber device (Fig. 4A). When using the haploid BY4742 and diploid BY4743 yeast strains, either a −0.2 V or −0.4 V vs. Ag/AgCl constant potential was applied to the microelectrode array for 24 h at RT in PBS(−). After 24 h, the electrode was washed with PBS(−) at RT, and the yeast cells attached to the electrode were observed using a phase-contrast microscope (CKX31, Olympus, Tokyo, Japan). After removing the PBS(−), 1 μL of YM medium was added to the ITO microelectrode regions and then incubated for 2 days at 28ºC under saturated water vapor conditions. After 2-day cultivation, the yeast cells proliferating on the microelectrode were transferred to 15-ml centrifuge tubes. The yeast cells in the centrifuge tubes were cultured with shaking (140 rpm) at 28ºC for a further 3 days.

### Optical microscopic observation

Viability of the yeast cells on the electrode was examined by analyzing the membrane permeability changes with a live/dead backlight bacterial viability kit for microscopy and quantitative assays according to the manufacturer's recommendations (L7012, Molecular Probes, Eugene, OR, USA). To obtain dead yeast cells to act as a benchmark, we cultured yeast cells with 1% (v/v) Tween 20 (Wako, Osaka, Japan) containing PBS(−) for 60 min at 60ºC. After constant potential application, the yeast cells on the patterned working electrode were washed with 25 ml of PBS(−) and stained with the live/dead backlight bacterial viability kit for 20 min at RT (Fig. 1). After incubation for 20 min, the patterned working electrode was again washed with 25 ml of PBS(−) and observed using an epifluorescence microscope system (BX51, Olympus, Tokyo, Japan) connected to a digital camera (DP72, Olympus) and image analysis system software (DP2-BSW, Olympus).

For the measurement of yeast cell density on the patterned working electrode, the numbers of yeast cells on the electrodes were counted in random areas of 100 × 100 μm^2^ after potential application. Measurement of yeast cell density was repeated eight times, and data were expressed as the mean of two independent experiments.

### Statistical analysis

Statistical analysis was performed using Student's *t*-test. The calculations were performed using Microsoft Excel.

### Scanning electron microscopic observation

*Saccharomyces cerevisiae* cells attached to the patterned ITO/glass electrode were prefixed with 2.5% glutaraldehyde in PBS(−) for 1 h at 28ºC. After washing with PBS(−) three times for 10 min each, the microorganisms were post-fixed with 2% osmium tetraoxide in PBS(−) for 2 h at 4ºC. After washing with distilled water at 4ºC six times for 10 min each, conductive staining was performed by incubation with 0.2% aqueous tannic acid (pH 6.8) at 4ºC for 30 min. The cells were washed with distilled water at 4ºC six times for 10 min each and then treated with 1% aqueous osmium tetraoxide at 4ºC for 30 min. After washing with distilled water at 4ºC six times for 10 min each, the cells were dehydrated in a graded ethanol series and critical point-dried **(**JCPD-5 critical-point drier, Japan Electron Optics Laboratories Ltd, Tokyo, Japan). The cells on the electrode were coated with osmium using an osmium plasma coater (POC-3, Meiwa Shoji Co., Osaka, Japan) and observed with a field-emission scanning electron microscope (SEM; JSM-6700F, Japan Electron Optics Laboratories Ltd) at an acceleration voltage of 5 kV.

## RESULTS

### Electrical attachment of *S. cerevisiae* to the patterned ITO electrode

To examine whether the yeast cells were attached to a reticulated ITO/glass electrode region with an applied potential (Fig. 1), we used both haploid and diploid strains of budding *S. cerevisiae* as test cells (Table 1). Figure 1C shows the distribution pattern of *S. cerevisiae* on the patterned ITO electrode after 24 h of constant potential application. A constant potential between +0.6 and −0.6 V vs. Ag/AgCl was applied to the patterned ITO electrode in PBS(−) for 24 h at RT. The *S. cerevisiae* diploid strain BY4743 was selectively attached to the reticulated ITO electrode surface to which a negative potential between −0.2 and −0.4 V vs. Ag/AgCl was applied (Fig. 1C). A −0.4 V vs. Ag/AgCl constant potential application induced the maximum attachment of the living diploid strain BY4743 cells to the reticulated ITO electrode region (Fig. 1C). Few or no diploid BY4743 cells selectively attached to the square glass regions (Fig. 1C). We observed that −0.5 V and −0.6 V vs. Ag/AgCl potentials occurred with electrical currents of −3.1 and −4.2 μA cm^−2^ due to the adsorption wave of positive ions in PBS(−) on the electrode surface and induced yeast cell membrane damage (Fig. 1). When we performed the cell viability test in the diploid BY4743 strain, it was confirmed that 29% (161 of 546 cells) and 6% (30 of 520 cells) of the cells remained alive after −0.5 V and −0.6 V vs. Ag/AgCl potential applications in PBS(−) for 24 h at RT, respectively (Fig. 1). The same phenomenon was observed in electrical retrievals of sediment and soil microorganisms (Koyama *et al.* 2013). We did not find any reduction in cell viability between −0.4 V and +0.6 V vs. Ag/AgCl potential applications in PBS(−). The haploid *S. cerevisiae* strains BY4741 and BY4742 were attracted by and selectively attached to the reticulated ITO electrode surface to which a potential between +0.6 and −0.3 V vs. Ag/AgCl was applied (Fig. 1C). The BY4741 haploid strain was prevented from attaching to the ITO electrode region with −0.4 V vs. Ag/AgCl constant potential application, while the BY4742 haploid strain non-specifically attached to both the ITO and square glass regions (Fig. 1C). Although most microorganisms including *S. cerevisiae* strains have a negative zeta potential at neutral pH (Nakari-Setälä *et al.* 2002; Schwegmann, Feitz and Frimmel 2010), only the diploid yeast strain BY4743 attached to the negative potential-applied ITO electrode region (Fig. 1C). The results in Fig. 1C suggest that the *S. cerevisiae* diploid strain BY4743 is selectively attracted to the negative potential-applied electrode surface.

Next, we investigated whether the yeast cells attached to the ITO electrode against gravitational force (Fig. 2A). Neither the haploid nor diploid strain of *S. cerevisiae* attached to the ITO electrode placed on the top of the three-electrode chamber device (Fig. 2A). To confirm accurate cell arrangement on the electrode surface, we examined whether *S. cerevisiae* diploid cells recognized small regions of the negative-applied potential microelectrode (Fig. 2B). Figure 2B shows *S. cerevisiae* diploid BY4743 cells attached to small regions of the patterned ITO electrode to which a negative potential was applied at RT in PBS(−). We confirmed that a small number of diploid BY4743 cells selectively attached to the negative potential 30 × 30 μm^2^ microelectrode array even if the electrode surface area was small compared with the reticulated electrode (Figs 1C and 2B). Few or no diploid BY4743 cells were attached to the SiO_2_-coated region of the microelectrode (Fig. 2B). Next, we examined the time course of *S. cerevisiae* attachment to the reticulated ITO electrode region (Fig. 2C). In the haploid strain of BY4742, the −0.2 V vs. Ag/AgCl applied potential induced cell attachment that increased in a linear fashion until 8 h, after which the cell attachment rate slowed (Fig. 2C). Meanwhile, attachment of the diploid BY4743 strain to the −0.4 V vs. Ag/AgCl-applied ITO electrode started from 5 h, and the cell density slowly increased compared with that of the BY4742 strain (Fig. 2C). The haploid and diploid cells attached to the bottom ITO electrode were observed using a SEM from a side-view angle of 75º (Fig. 2D). Both the haploid BY4742 and diploid BY4743 strains of *S. cerevisiae* appeared to adhere directly to the negative potential-applied ITO electrode surface (Fig. 2D).

To clarify the interaction between the diploid yeast strain BY4743 and the negative applied-potential electrode, we examined whether the diploid yeasts were attracted by and attached to a transparent GZO/glass electrode surface instead of the ITO electrode (Fig. 3). The resting potential of the GZO electrode in PBS(−) at RT was −284 ± 10 mV vs. Ag/AgCl (mean ± SEM; *n* = 12) and was lower than that of the ITO electrode (+5 ± 5 mV vs. Ag/AgCl; *n* = 104). Therefore, we observed the distribution pattern and cell density of *S. cerevisiae* diploid BY4743 cells on the patterned GZO electrode after 24 h of constant potential applications between +0.6 and −0.4 V vs. Ag/AgCl (Fig. 3). Few or no diploid BY4743 yeast cells were attached to the GZO electrode to which potentials between +0.6 and −0.4 V vs. Ag/AgCl were applied (Fig. 3). The results in Figs 1–3 indicate that the *S. cerevisiae* diploid strain BY4743 cells were selectively attracted to the negative potential-applied ITO electrode surface.

### Proliferation of single *S. cerevisiae* cells attached to the ITO microelectrode

All of the yeast cells attached to the ITO electrode surface were easily detached by scraping several times with the rubber cell scraper. Therefore, we compared yeast cell growth after detaching from both a negative potential-applied electrode and a resting potential (OC)-applied electrode (Fig. 2E). No statistically significant difference in cell growth was observed in the negative potential- and the resting potential-applied haploid BY4742 and diploid BY4743 yeast cells, respectively (Fig. 2E). A 1-h delay was seen in the growth of the potential-applied BY4742 and BY4743 strains after 24 h of PBS(−) treatment compared with the subcultures of the two strains (Fig. 2E).

Next, we examined whether single yeast cells attached to small regions of the negative applied-potential microelectrode could proliferate (Fig. 4). The ITO microelectrode array was arranged in 16 rows and 16 lines with a distance of 4.5 mm between them to allow manipulation of the cells and medium with a commercially available multichannel micropipette (Fig. 4A and B). We confirmed that a small number of haploid and diploid yeast cells selectively attached to the negative potential-applied 50 × 50, 100 × 100, and 200 × 200 μm^2^ microelectrode arrays (Fig. 4C and D). After applying a −0.4 V vs. Ag/AgCl potential and removal of PBS(−), we added 1 μL of YM medium to each ITO microelectrode region and then the yeast cells were incubated for 2 days at 28ºC (Fig. 4B and C). We confirmed the growth of the attached diploid BY4743 single yeast cells after 2 days on the microelectrode and after 5 days in tube culture (Fig. 4B and C). The results in Figs 2 and 4 clearly show that the spatial configuration and cell regrowth of diploid single yeast cells were successful using the patterned ITO microelectrode culture system.

### The flocculin family is involved in the cell–ITO surface interaction

**Figure 5.**
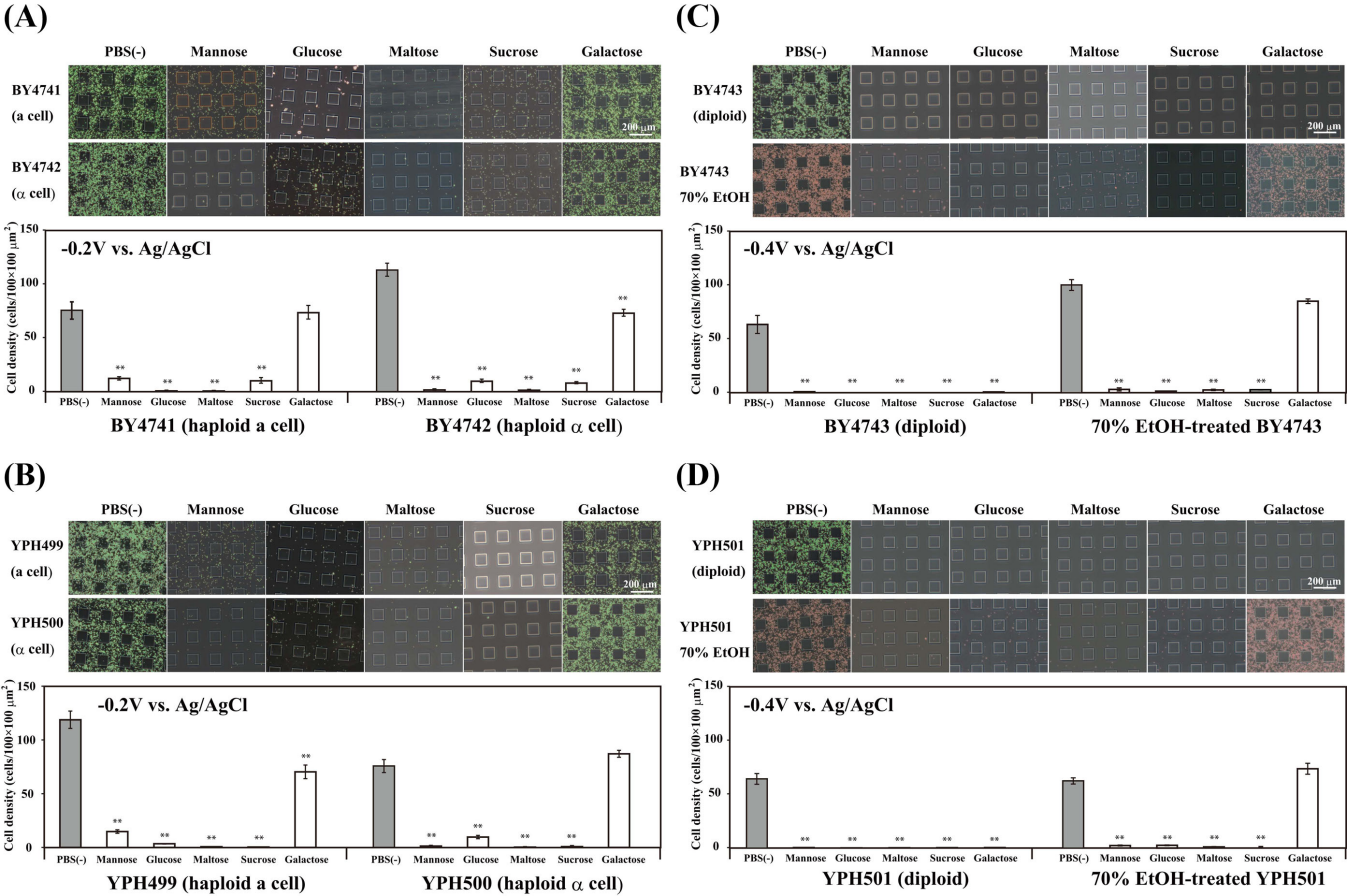
Inhibitory effects of five sugar solutions on *S. cerevisiae* adhesion to the negative potential-applied ITO electrode surface. (**A**) BY4741 and BY4742 haploid strains. (**B**) YPH499 and YPH500 haploid strains. (**C**) BY4743 diploid strain. (**D**) YPH501 diploid strain. The BY4743 and YPH501 strains pretreated with 70% EtOH for 1 h at 60ºC were stained with propidium iodide alone. Either an OC, a −0.2 V vs. Ag/AgCl constant potential, or −0.4 V vs. Ag/AgCl constant potential was applied for 24 h in PBS(−) or in each of the sugar solutions (all 280 mM) at RT, respectively. The values are mean ± SEM (*n* = 8). **P < 0.001 compared with PBS(−).

Our results shown in Figs 1–4 indicated that the *S. cerevisiae* diploid strain BY4743 was selectively attracted to the negative potential-applied ITO electrode surface. Possible adhesion proteins in *S. cerevisiae* are specialized cell-surface proteins called the flocculin family which bind sugar residues on the surface of other cells or promote binding to abiotic surfaces (Guo *et al.* 2000; Reynolds and Fink 2001; Verstrepen and Klis 2006; Mortensen *et al.* 2007; Van Mulders *et al.* 2009). Flocculation phenotypes of the flocculin family can be divided into two groups: the Flo1-phenotype such as the Flo1 protein and the NewFlo-phenotype such as the Flo10 protein (Stratford and Assinder 1991; Goossens and Willaert 2010). This grouping is based on the type of carbohydrate which inhibits flocculation. Flocculation of the Flo1 protein can be inhibited by mannose, but not by glucose, maltose, sucrose or galactose (Stratford and Assinder 1991; Van Mulders *et al.* 2009; Goossens and Willaert 2010). Flo10 flocculation can be inhibited by mannose, glucose, maltose and sucrose, but not by galactose (Van Mulders *et al.* 2009). We examined whether five sugar solutions of mannose, glucose, maltose, sucrose and galactose inhibited the attachment of *S. cerevisiae* to the ITO electrode. Figure 5 shows the inhibitory effects of the five sugar solutions on *S. cerevisiae* adhesion to the negative potential-applied ITO electrode surface. The attachment of the four haploid strains BY4741, BY4742, YPH499 and YPH500 to the −0.2 V vs. Ag/AgCl-applied ITO electrode for 24 h was significantly inhibited by mannose, glucose, maltose and sucrose, while no or only slight inhibition was observed with galactose (Fig. 5A and B). The attachment of both the BY4743 and YPH501 diploid strains to the electrode was almost completely inhibited by all five sugar solutions (Fig. 5C and D). When diploid BY4743 and YPH501 strains were pretreated with 70% EtOH for 1 h at 60ºC, no or only very slight inhibition of attachment to the −0.4 V vs. Ag/AgCl-applied ITO electrode was seen in galactose solution, while mannose, glucose, maltose and sucrose significantly inhibited the adhesion of the EtOH-fixed diploid strains to the negative potential-applied ITO electrode (Fig. 5C and D). The results in Fig. 5 suggest that the mechanisms of *S. cerevisiae* cell adhesion to the negative potential-applied ITO electrode might involve the Flo10 proteins, which can be inhibited by mannose, glucose, maltose and sucrose, but not by galactose (Van Mulders *et al.* 2009).

**Figure 6.**
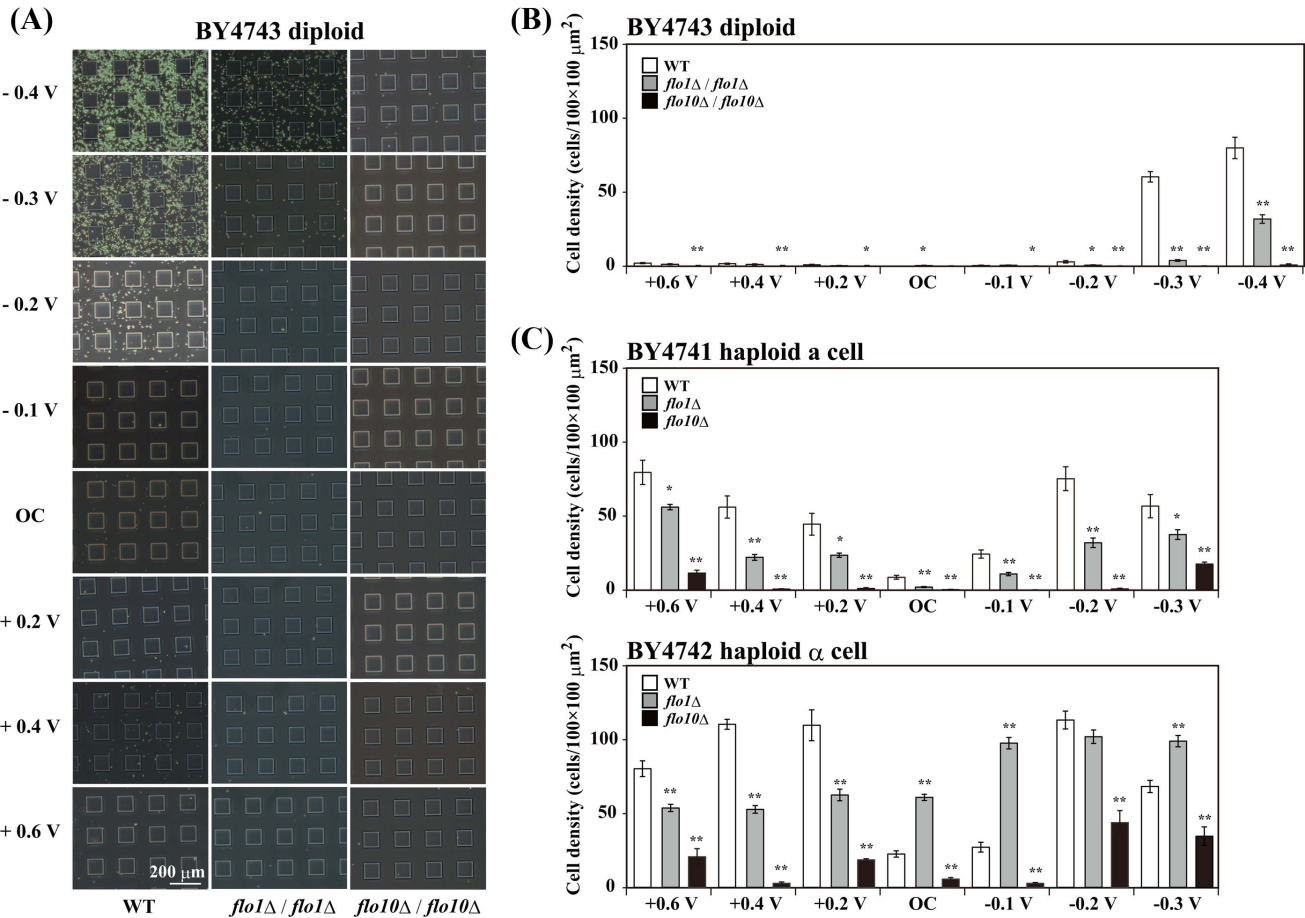
*FLO1* and *FLO10* gene-disrupted *S. cerevisiae* strains on the potential-applied ITO electrode. (**A**) Distribution patterns of wild-type (WT), *FLO1* gene-disrupted (*flo1*Δ/ *flo1*Δ) and *FLO10* gene-disrupted (*flo10*Δ/ *flo10*Δ) BY4743 diploid strains on the ITO electrode to which a potential between −0.4 V and +0.6 V vs. Ag/AgCl was applied for 24 h in PBS(−) at RT. (**B**) Cell density of wild-type (WT), *FLO1* gene-disrupted (*flo1*Δ/ *flo1*Δ) and *FLO10* gene-disrupted (*flo10*Δ/ *flo10*Δ) BY4743 diploid strain on the potential-applied ITO electrode. (**C**) Cell density of wild-type (WT), *FLO1* gene-disrupted (*flo1*Δ) and *FLO10* gene-disrupted (*flo10*Δ) BY4741 and BY4742 haploid strains on the potential-applied ITO electrode. The values are mean ± SEM (*n* = 8). *P < 0.05, **P < 0.001 compared with WT.

We then investigated whether either the *FLO1* or *FLO10* gene-disrupted *S. cerevisiae* mutants were able to attach to the negative potential-applied ITO electrode. Figure 6A and B shows the distribution pattern and cell density of diploid BY4743 wild-type, *flo1*Δ/*flo1*Δ and *flo10*Δ/*flo10*Δ mutants on the patterned ITO electrode after 24 h of constant potential application. The *flo10*Δ/*flo10*Δ mutant showed almost complete loss of attachment to the ITO electrode to which a potential between +0.6 and −0.4 V vs. Ag/AgCl was applied (Fig. 6A and B). The inhibition of the attachment of the BY4743 *flo1*Δ/*flo1*Δ mutant to the negative potential-applied ITO electrode was statistically significant compared with that of the wild-type strain (Fig. 6A and B). The cell density of the *flo10*Δ mutants of both the haploid BY4741 and BY4742 strains was decreased on the ITO electrode to which a potential between +0.6 and −0.3 V vs. Ag/AgCl was applied (Fig. 6C). Irrespective of potential application, the attachment of the BY4741 *flo1*Δ mutant to the ITO electrode was significantly less than that of the wild-type strain, while the BY4742 *flo1*Δ mutant showed a tendency to attach to the ITO electrode (Fig. 6C). The results in Figs 5 and 6 clearly show that the *FLO10* gene strongly conferred the ability to attach to the negative potential-applied ITO electrode on the *S. cerevisiae* strains

## DISCUSSION

Flocculation of yeasts has been studied with *S. cerevisiae*, *S. uvarum*, *Kluyveromyces bulgaricus*, *K. lactis* and *K. marxianus* (Miki *et al.* 1982; Hussain *et al.* 1986; Bellal *et al.* 1995; Almeida *et al.* 2003). In general, native and commercial yeasts are non-flocculent (Nonklang *et al.* 2009; Govender, Bester and Bauer 2010). *Saccharomyces cerevisiae* flocculation has been defined as the asexual, reversible and calcium-dependent aggregation of yeast cells to form flocs containing thousands of cells which rapidly sediment to the bottom of liquid growth substrate (Stratford 1989; Bony *et al.* 1997). Structural and functional analysis of the genomic sequence of *S. cerevisiae* reveals that this yeast includes the five distinct flocculin family genes *FLO1*, *FLO5*, *FLO9*, *FLO10* and *FLO11* (Teunissen and Steensma 1995; Caro *et al.* 1997). The *FLO1*, *FLO5*, *FLO9* and *FLO10* genes confer cell–cell adhesion (flocculation) ability, and only the *FLO11* gene does not contain the mannose-binding PA14 domain (Goossens and Willaert 2010, 2012) and is responsible for adhesion to substrates such as plastic and agar (Guo *et al.* 2000; Verstrepen and Klis 2006; Mortensen *et al.* 2007).

The haploid *S. cerevisiae* BY4741 and BY4742 strains adhered not only to the negative potential-applied ITO electrode surface but also positive potential-applied ITO electrode surface (Figs 1 and 6). Cell adhesion of the four haploid strains to the −0.2 V vs. Ag/AgCl potential-applied ITO electrode was inhibited in the presence of sucrose, mannose, maltose and glucose, but not in the presence of galactose (Fig. 5). The *FLO1* or *FLO10* gene-disrupted haploid strains showed partially inhibited cell attachment to the ITO electrode surface to which a potential between +0.6 and −0.3 V vs. Ag/AgCl was applied (Fig. 6). Therefore, the PA14 domains of *FLO1*, *FLO5*, *FLO9* and/or *FLO10* may contribute to the competitive recognition of the ITO electrode surface by the yeast strains.

The living diploid *S. cerevisiae* strains BY4743 and YPH501 showed almost completely inhibited attachment to the −0.4 V vs. Ag/AgCl potential-applied ITO electrode surface in the presence of sucrose, mannose, maltose, glucose and galactose solutions (Fig. 5). However, the BY4743 and YPH501 strains pretreated with 70% EtOH attached to the negative potential-applied ITO electrode in the presence of galactose (Fig. 5). In the final stage of beer fermentation, yeast cells clump together and form a sediment at the bottom of the fermentation tank when all fermentable sugars are converted into ethanol and carbon dioxide (Van Mulders *et al.* 2010). Therefore, the attachment of strain BY4743 to the ITO electrode would be controlled by the presence or absence of sugars in the surrounding environment.

The diploid BY4743 cells did not attach to the GZO electrode but adhered to the negative potential-applied ITO electrode (Fig. 3). Moreover, the BY4743 mutant (*flo10*Δ/*flo10*Δ) lost the ability to attach to the ITO electrode (Fig. 6). Therefore, most of the flocculin proteins on the cell wall of the BY4743 strain would be the Flo10 protein, which interacts with the negative potential-applied ITO electrode surface. The alignments of the *FLO5*, *FLO9* and *FLO10* genes with the *FLO1* gene show similarities of 96, 94 and 58%, respectively (Teunissen and Steensma 1995). The major differences between the *FLO1* and *FLO10* genes are found in the middle domain (Teunissen and Steensma 1995). It is believed that calcium ions stabilize the middle domain of the flocculin proteins and allow them to take their active conformation (Miki *et al.* 1982; Stratford and Assinder 1991; Guo *et al.* 2000; Verstrepen and Klis 2006; Soares 2010). The negative potential-applied ITO electrode with sodium and/or potassium ions in PBS(−) may stabilize the middle domain of the Flo10 proteins on the cell wall surface of strain BY4743 and activate the interaction between the Flo10 proteins and the negative potential-applied ITO electrode.

In this study, we demonstrated that *S. cerevisiae* selectively attached to ITO electrode surface regions. Moreover, our results indicate that the mechanisms of *S. cerevisiae* diploid strain BY4743 adhesion involve interaction between the negative potential-applied ITO electrode surface and flocculin Flo10 proteins on the cell wall surface. The *FLO10* gene expressed in *S. cerevisiae* on the negative potential-applied ITO electrode can easily be replaced by a chemical compound in the medium. Therefore, the heterozygous diploid mutant single *S. cerevisiae* cells attached to the ITO microelectrode array will allow iterative screening tests of numerous pharmaceutical compound candidates. Moreover, the expression of Flo10 proteins in non-flocculent yeast strains would also be useful in fermentation processes because the yeast strains expressing Flo10 can be separated easily from the fermentation mash.
